# Oral versus Long-Acting Injectable Antipsychotics in the Treatment of Schizophrenia and Special Populations at Risk for Treatment Nonadherence: A Systematic Review

**DOI:** 10.1155/2012/407171

**Published:** 2012-02-15

**Authors:** Simon Zhornitsky, Emmanuel Stip

**Affiliations:** Département de Psychiatrie, Université de Montréal, Montréal, Québec, Canada 2900

## Abstract

Long-acting injectable antipsychotics (LAIs) should offer better efficacy and tolerability, compared to oral antipsychotics due to improved adherence and more stable pharmacokinetics. However, data on LAIs has been mixed, with some studies finding that they are more effective and tolerable than oral antipsychotics, and others finding the contrary. One possibility for the disparate results may be that some studies administered different antipsychotics in the oral and injectable form. The present systematic review examined the efficacy and tolerability of LAIs versus their oral equivalents in randomized and naturalistic studies. In addition, it examined the impact of LAIs on special populations such as patients with first-episode psychosis, substance use disorders, and a history of violence or on involuntary outpatient commitment. Randomized studies suggest that not all LAIs are the same; for example, long-acting risperidone may be associated with equal or less side effects than oral risperidone, whereas fluphenazine decanoate and enanthate may be associated with equal or more side effects than oral fluphenazine. They also suggest that LAIs reduce risk of relapse versus oral antipsychotics in schizophrenia outpatients when combined with quality psychosocial interventions. For their part, naturalistic studies point to a larger magnitude of benefit for LAIs, relative to their oral equivalents particularly among first-episode patients.

## 1. Introduction

Schizophrenia is a severely disabling psychiatric disorder that often includes hallucinations, delusions, cognitive deficits, poor insight, and comorbid substance use disorder (SUD) [1–3]. The first decade of illness in schizophrenia patients is often characterized by repeated episodes of psychosis with varying levels of remission between episodes and increased disability following each episode [3–5]. Moreover, the bulk of functional deterioration tends to occur in the first five years after onset of schizophrenia, after which the illness typically progresses into a stable phase, wherein positive symptoms are decreased and negative, and cognitive symptoms become predominant [3].

Antipsychotics—drugs that block dopamine D_2_ receptors—attenuate positive symptoms in schizophrenia and help improve outcomes, especially in the early stages of illness [4, 6–8]. For example, a pivotal study by May [8] revealed that treatment with antipsychotics or electroconvulsive therapy increased the rate of release from the hospital, reduced the length of hospital stay, and decreased the need for sedatives and hydrotherapy in newly admitted first-episode psychosis (FEP) patients, relative to psychotherapy or milieu therapy. Another study by Crow et al. [6] showed that 46% of 120 FEP patients maintained on antipsychotics relapsed within two years, compared to 62% of patients on placebo. Importantly, the chance of subsequent relapse was significantly increased in patients with a longer duration of untreated psychosis. Overall, multiple reviews found that that early antipsychotic treatment is crucial for improving outcomes in FEP patients and may prevent some functional deterioration and development of a chronic course [4, 5].

Despite the benefits of compliance with antipsychotic therapy during the early stage of illness, data from 2,588 FEP patients revealed that only 58% collected their prescription during the first 30 days of hospital discharge, and only 46% continued their initial treatment for 30 days or longer [9]. Indeed, studies have consistently shown that more than 40% of patients with FEP are nonadherent and discontinue medication during the first nine months of treatment, at which point the chances of relapse increase dramatically [10, 11]. The high rate of noncompliance can be explained by poor insight into illness, cognitive deficits, and elevated substance abuse associated with schizophrenia and by side effects associated with antipsychotics such as anhedonia and extrapyramidal symptoms (EPS) [3]. Clearly, the goal of treatment of FEP patients should be to increase compliance with antipsychotic therapy, thereby decreasing the negative effects of untreated psychosis and—at the same time—to minimize the amount of antipsychotic-induced side effects. This goal may, in part, be achieved by the use of long-acting or depot antipsychotics (LAIs) [12–14].

LAIs have a number of advantages over oral antipsychotics. First, they should decrease noncompliance due to forgetfulness and loss of insight (e.g., due to psychotic relapse or substance abuse) because patients are followed up if they miss an appointment for their injection [15]. Moreover, LAIs should maximize pharmacokinetic coverage and minimize antipsychotic withdrawal symptoms resulting from partial compliance [16]. In addition, LAIs are not influenced by first-pass metabolism, decreasing the potential for drug-drug interactions. Finally, some have argued that because the slow rate of absorption associated with LAIs leads to reductions in differences between C_max⁡⁡_ (peak) and C_min⁡⁡_ (trough) plasma levels [17], they should induce less side effects (an important predictor of poor treatment compliance), relative to oral antipsychotics [18].

Older (typical) LAIs were made by forming an ester with a fatty acid such as decanoic acid and injecting it in an oily solution such as sesame seed oil or low viscosity vegetable oil, thereby maximizing its lipophilicity and affinity for fatty tissue [19]. Moreover, the resulting highly lipophilic compounds possess more complicated, multicompartment tissue binding [28]. Oil-based formulations must be injected slowly and are commonly associated with acute injection site pain, and skin reactions that can last for up to three months [20, 22]. They may induce “breakthrough EPS” on the day of the injection, which is caused by a small amount of free drug released immediately into the patient's system [29–33]. They may also be detectable in plasma for as long as six months following an injection, increasing the possibility of interaction with other drugs [34–36]. By contrast, some of these phenomena should be minimized with the atypical, water-based LAIs, including risperidone microspheres, and olanzapine pamoate (Table 1) [24, 31, 37, 38].

Interestingly, studies on efficacy of LAIs versus oral antipsychotics have produced conflicting results. For example, a 2-year study by Rosenheck et al. [39] revealed that risperidone LAI did not significantly decrease relapse over mixed oral antipsychotics, but it produced more EPS in unstable schizophrenia patients. Our own 2-year, randomized trial among FEP in/outpatients did not find any efficacy or tolerability differences between risperidone LAI and mixed oral atypical antipsychotics; however, the former medication was associated with a reduction in noncompliance [40]. Altogether, a meta-analysis by Adams et al. [41] did not find major efficacy differences between LAIs and oral antipsychotics in randomized studies of schizophrenia inpatients and outpatients. However, in these studies, the combination of inpatients with outpatients may have biased results towards oral antipsychotics, since medication compliance should be more strictly controlled in an inpatient setting [42]). More recently, Leucht et al. [43] showed LAIs to be superior to oral antipsychotics for preventing relapse among schizophrenia outpatients only.

Although the limitation of including both inpatients and outpatients was addressed by Leucht et al. [43], there is still the confound of comparing different molecules in the oral and injectable forms—an effect that may have confounded results due to the fact that not all antipsychotics possess an equal efficacy and tolerability profile [44–46]. The present systematic review will examine the efficacy and tolerability of oral and injectable forms of the same antipsychotic in randomized studies. However, since noncompliant patients are unlikely to participate in randomized studies, we will also include naturalistic studies administering LAIs to a general population of schizophrenia patients and to special populations at risk for treatment nonadherence such as patients with FEP, SUDs, and those with a history of violence or on involuntary outpatient commitment. 

## 2. Methods

A systematic search was carried out in the electronic databases, PubMed and EMBASE, using the keywords “antipsychotic” and “depot” or “injectable” and “randomized” or “naturalistic” or “first-episode” or “noncompliance” or “substance abuse” or “substance use disorder,” or “alcohol” or “drug” or “cannabis,” or “cocaine” or “heroin” or “amphetamine” or “violence” or “involuntary outpatient commitment” or “community treatment order.” This search looked for studies published between 1 January 1960 and 1 July 2011. In addition, studies and published abstracts were identified by cross-referencing of review articles. Unpublished studies were identified using clinicaltrials.gov.

For our analyses of randomized (open-label and double-blind) and naturalistic studies in the general population of schizophrenia patients, we included only trials comparing an LAI with its oral equivalent. However, due to a lack of studies, we included any comparative pharmacological trial in our examination of LAI-treatment of individuals with FEP, SUDs, and those on involuntary outpatient commitment. Mirror-image studies were excluded. Due to the fact that there were no studies investigating paliperidone LAI versus oral paliperidone, it was not included in the analyses. 

## 3. LAIs Versus Oral Equivalent—Randomized Studies

Studies comparing an LAI with its oral equivalent in hospitalized patients are conducted to show the “non-inferior” efficacy and/or to study differences in tolerability and pharmacokinetics of the formulations. Hospitalized patients are useful for these purposes because compliance with oral antipsychotics should be maximized in an inpatient setting, limiting the potential confounding effect of noncompliance. By contrast, studies of LAIs versus oral antipsychotics conducted in outpatients are designed to mimic the “real world” setting where compliance is a problem.

### 3.1. Haloperidol Decanoate

One four-month study among schizophrenia inpatients revealed that haloperidol decanoate was associated with marginally better efficacy and more EPS, relative to oral haloperidol (Table 2) [47].

### 3.2. Fluphenazine Enanthate and Decanoate

Four short-term studies compared the efficacy and tolerability of fluphenazine enanthate versus oral fluphenazine among inpatients. All of the studies found that fluphenazine enanthate was equivalent to oral fluphenazine for schizophrenia symptoms [48–51]; however, two of the studies also showed that the former produced more EPS than the latter [50, 51]. Interestingly, in the Van Praag et al. [51] study, a marked increase in EPS was witnessed on the week of each injection, and it decreased somewhat the following week, which may be evidence of “breakthrough EPS.” Pharmacokinetic studies have shown that fluphenazine plasma levels spike after administration of the decanoate and enanthate formulations [29, 30]. However, plasma levels of the latter decline much more slowly than that of the former, which may be another reason why two of the aforementioned studies found that fluphenazine enanthate was associated with more EPS overall.

Four pivotal, long-term studies compared fluphenazine enanthate and decanoate with their oral equivalent in stabilized outpatients [52–55]. A 21-month study by Del Giudice et al. [52] showed that fluphenazine enanthate was superior to the oral fluphenazine in reducing relapse among 82 schizophrenia outpatients. Analysis of tolerability outcomes revealed that 6 patients on fluphenazine enanthate experienced EPS, whereas none experienced EPS on oral fluphenazine. The design of this study was unique because a nurse was available to administer injections on home visits, which may have increased compliance with injections and reduced relapse. On the other hand, the short length of her visits may have led to underreporting of side effects, since fluphenazine levels may take days to peak following administration of the enanthate [29, 30, 52]. Accordingly, comparisons between the two esters showed that the decanoate produces the majority of EPS up to nine hours after the injection, whereas the enanthate produces it 12–48 hours after the injection [62].

Fluphenazine decanoate was compared to its oral equivalent in a study by Rifkin et al. [53]. The authors found that both treatments were equally superior to placebo in decreasing 1 year relapse rates among 73 outpatients. However, there were significantly more terminations due to toxicity in the fluphenazine decanoate compared with the oral group, and this was attributed to the fact that 35% of patients receiving the former treatment developed severe akinesia. Akinesia was measured by the BPRS and included such items as emotional withdrawal, depressed mood, blunted effect as well as motor retardation [63]. Importantly, the authors noted that patients on oral fluphenazine were relatively reliable pill takers (as defined by pill counting and urine tests)—a fact that renders the efficacy results fairly meaningless.

Hogarty et al. [54] conducted an important two-year study in 105 schizophrenia outpatients. The authors demonstrated that fluphenazine decanoate decreased relapse, but only when combined with intensive individual and family social therapy. In fact, no patient relapsed in the group treated with fluphenazine decanoate and social therapy after month eight, until the end of the study. Interestingly, there was no impact of social therapy in patients treated with oral fluphenazine. Moreover, analyses revealed that fluphenazine decanoate was associated with significantly more symptoms of depression and anxiety, whereas fluphenazine hydrochloride was associated with more positive symptoms [54]. A larger study by Schooler et al. [55] among 214 schizophrenia outpatients did not find a difference in relapse or side effects between fluphenazine decanoate and oral fluphenazine in schizophrenia outpatients over a period of 1 year. However, the oral equivalent mean dose was higher (25 mg/d), and the decanoate dose was lower (34 mg/3 w) than in some previous studies, which makes interpretation difficult [55]. Taken together, the aforementioned studies suggest that fluphenazine decanoate and enanthate may be associated with similar or more side effects than oral fluphenazine. This finding could be explained by the initial spikes in plasma levels as well as decreased conversion of fluphenazine to its clinically inactive sulfoxide metabolite [30, 64]. Additionally, the results may be explained by the fact that more patients are assumed to be noncompliant in the oral fluphenazine group, and thus to experience less side effects (but see [53]). Concerning efficacy, the studies suggest that there is a benefit of injectable fluphenazine preparations in reducing time to relapse among schizophrenia outpatients when combined with additional interventions such as intensive social therapy and/or a nurse available for home visits. 

### 3.3. Zuclopenthixol Decanoate

The only study involving zuclopenthixol was a 1-year study in 46 schizophrenia outpatients with previous violence [56]. The study demonstrated that schizophrenia patients on zuclopenthixol decanoate reduced the number of violent episodes per month of the study and increased the number of months of adherence to medication. Moreover, the authors found that PANSS positive scores were nonsignificantly lower in decanoate-treated patients. However, it is possible that the lack of difference in symptoms may be a type-II error since these patients also had significantly higher PANSS-positive scores at baseline, relative to patients treated with the oral formulation (despite randomization) [56].

### 3.4. Risperidone Microspheres

Long-acting risperidone was compared with its oral equivalent in two randomized trials. A 12-week study among 541 inpatients and outpatients found no efficacy difference, but risperidone LAI (25 mg, 50 mg or 75 mg/2 w) produced significantly less prolactin elevation than oral risperidone (2 mg, 4 mg, or 6 mg/d) [26]. Similarly, a 48-week study by Bai et al. [57] in 50 inpatients revealed no significant differences in overall efficacy; however, long-acting risperidone (25 mg, 37.5 mg, or 50 mg/2 w) produced significantly less EPS and prolactin elevation and lower serum concentrations of 9-hydroxy-risperidone (9-OH risperidone, paliperidone), relative to oral risperidone (4 mg, 5-6 mg, or 7+ mg/d) [57]. However, subanalyses revealed that patients who received the two lowest doses showed increased PANSS scores and an increased tendency to relapse. Taken together, these data reveal that risperidone LAI may produce equal or less side effects, compared to oral risperidone. The data may be explained by the fact that treatment with long-acting risperidone leads to significantly lower serum concentrations of risperidone and 9-OH risperidone [65, 66]—the latter which also displays high affinity for D_2_ receptors [67]. They may also be explained by the lack of “breakthrough EPS” and depressive symptoms associated with long-acting risperidone, in contrast to fluphenazine LAIs. Finally, the differences may be explained by the difficulty in finding equivalent doses between LAIs and oral antipsychotics. Interestingly, Bai et al. [57] found that 25 mg and 37.5 mg/2 w provided insufficient efficacy when switching from 4 mg and 5-6 mg/d oral risperidone, respectively. Based on their results, the authors recommended that the threshold for switching should be reduced to oral risperidone 3 mg/d for risperidone LAI 37.5 mg/2 w, and oral risperidone 5 mg/d for risperidone LAI 50 mg/2 w.

### 3.5. Olanzapine Pamoate

An unpublished, open-label, 2-year study among 524 outpatients evidenced that those treated with olanzapine pamoate required significantly fewer hospitalizations; however, there were no other major differences in efficacy or side effects [58]. Moreover, a recent 24-week study has found similar efficacy between a high and a medium of olanzapine pamoate (300 mg/2 w; 405 mg/4 w) and oral olanzapine (10, 15, and 20 mg/d) in 1065 schizophrenia outpatients [25]. Patients treated with a low dosing regimen (150 mg/2 w) evidenced significantly more psychotic exacerbation, compared to those treated with the high dose. Olanzapine plasma levels in patients treated with the 405 mg/4 w dose were lower than that of patients maintained on the oral equivalent of 15 mg/d, and they evidenced (nonsignificantly) less weight gain (15%; criteria ≥ 7% of baseline) than patients treated with 300 mg/2 w (21%) and patients on 15 mg/d oral olanzapine (21%) [25], suggesting that this may be the optimal dosing to maintain high efficacy, without compromising tolerability, especially given the risk of inadvertent intravascular injections [24].

It is important to note that in most of the aforementioned randomized studies patients were selected on the basis of having been stabilized on (and likely adherent to) the oral equivalent prior to the study, resulting in a potential bias in favor of oral formulations [25]. This interpretation is consistent with results of Rifkin et al. [53], which found that patients on oral fluphenazine were, in fact, reliable pilltakers.

## 4. LAIs versus Oral Equivalent—Naturalistic Studies in Schizophrenia and FEP Patients

Naturalistic studies are an important indicator of the value of LAIs because they include patients for whom these agents are typically prescribed. Four naturalistic studies all found that LAIs were superior to their oral equivalents. Among the general population of schizophrenia patients, there is evidence that patients treated with haloperidol or fluphenazine LAI had a significantly longer mean time to all-cause medication discontinuation and were twice as likely to stay on medication, compared to patients treated with oral haloperidol or fluphenazine [59]. Among FEP patients, a 2-year study showed that risperidone LAI significantly reduced relapse and improved compliance, relative to oral risperidone [60]. Similarly, a cohort study of 2,234-consecutive FEP patients showed that perphenazine depot was associated with the lowest relative risk of rehospitalization, compared to oral perphenazine and other typical and atypical antipsychotics [61]. A more recent study by the same authors examined the risk of rehospitalization and drug discontinuation in 2,588 FEP patients [9]. The authors found that the risk of rehospitalization for FEP patients receiving LAIs was two thirds lower than for patients receiving the oral equivalent. These data support the notion that randomized studies in outpatients may underestimate the efficacy benefits of LAI therapy because they underrepresent noncompliant schizophrenia patients.

## 5. LAIs for SUDs

The lifetime prevalence of comorbid SUDs in schizophrenia patients is estimated to be nearly 50% [68]. Among FEP patients, a recent 2-year followup study found that 24% of individuals abused either alcohol or drugs at baseline and 72% of substance abusers, and 31% of nonabusers had experienced at least one occasion of involuntary hospitalization [69]. In general, studies suggest that compared to nonabusing patients, dual diagnosis schizophrenia patients have more psychiatric symptoms and EPS [70], they are more frequently hospitalized, suicidal, impulsive and violent, homeless, and unemployed, and they have more legal and health problems [71, 72]. Moreover, substance use is commonly associated with poor adherence to antipsychotic treatment, and, naturally, LAIs are commonly recommended as for improving adherence in psychosis patients with comorbid SUDs [14, 31, 73]. In order to better elucidate the potential implications of prescribing LAIs to SUD patients, we examined the literature for comparative studies in dual diagnosis patients. Due to the lack of LAI trials in this group, we also included randomized studies administering LAIs in nonpsychosis substance abusers.

Only one published study exists, which randomized dual diagnosis patients to treatment with an LAI. It was a 6-month, open-label trial that compared long-acting risperidone with zuclopenthixol decanoate in outpatients with mixed SUDs [74]. The authors found that schizophrenia patients treated with risperidone LAI evidenced significantly diminished substance use (measured by urine screens), less PANSS-negative symptoms, less EPS, and better compliance with a substance abuse treatment program. Unfortunately, however, all patients relapsed in both treatment groups [74]. In addition, a retrospective study compared the effectiveness of oral olanzapine, risperidone, ziprasidone, and typical LAIs in tobacco-dependant inpatients with mixed SUDs, undergoing a 90-day substance abuse treatment program [75]. Results revealed that significantly fewer patients treated with olanzapine and typical LAIs completed the program, relative to those treated with risperidone. Moreover, patients treated with oral olanzapine and typical LAIs stayed for a shorter time in treatment, and all claimed that they planned to smoke immediately after discharge, compared to only 56% for risperidone and 50% for ziprasidone [75]. Knowing that cigarette smoke induces the enzyme (CYP1A2) responsible for the metabolism of olanzapine and most typicals [76, 77], and that patients were forbidden to smoke during the program, it was hypothesized that individuals on olanzapine and typicals were noncompliant with treatment because they were experiencing more side effects due to rising plasma levels of the antipsychotics. Indeed, the authors noted subtle effects such as mild sedation that affected cognitive ability or motivation and led the patients to be recorded as being “sleepy” or “inattentive” [75].

Four studies randomized nonpsychosis SUD patients to treatment with an LAI, relative to placebo. A large 6-month trial in nonpsychosis alcoholics found that flupenthixol decanoate (10 mg) aggravated relapse to alcohol use, relative to placebo, possibly due to increased craving for alcohol [78]. Another study reported that long-acting risperidone had no effect on cocaine use or craving but significantly aggravated depressive symptoms, compared to placebo [79]. Two other studies evidenced high rates of EPS following the administration of flupenthixol decanoate in cocaine abusers [80–82]. Specifically, a pilot trial found that a large portion of individuals treated with flupenthixol decanoate (12 mg) experienced severely disturbing akathisia, dysarthria, myalgia, and dystonia that required medical intervention after they smoked crack cocaine [80, 81]. More recently, an experimental study by Evans et al. [82] has showed that flupenthixol decanoate (10 and 20 mg) dose dependently increased the desire for cocaine, drug liking, drug potency and good drug effects in cocaine abusers given intravenous injections of cocaine. The study was terminated by the sponsor after the second of seven subjects experienced a dystonic reaction in the high-dose flupenthixol group. Intriguingly, the latter two studies provide clinical evidence for the existence of dopaminergic supersensitivity to psychostimulant challenge—a consequence of repeated antipsychotic treatment in preclinical models [83].

Altogether, the preliminary studies on LAIs in psychosis and nonpsychosis substance abusers show the potential for these agents to aggravate of substance abuse, possibly through increased incidence of adverse events (anhedonia, EPS, sedation) and drug liking/craving. In comparison to typical LAIs, long-acting risperidone seems to produce better outcomes and may be helpful, especially when relapse and rehospitalization due to noncompliance is a concern. In addition, oil-based vehicle should be avoided for SUD patients because they are cleared from the system more slowly. Oil-based vehicles such as sesame seed oil and viscoleo may extend the half-life of antipsychotics by accumulating in tissue and prolonging absorption [84]. For example, there is evidence that six months after discontinuation of treatment with low-dose haloperidol decanoate (30–50 mg/4 weeks), D_2_ receptor occupancy reached 24%, 32% and 34% in three of four patients [85]. Likewise, other studies have reported persistently elevated plasma fluphenazine and prolactin levels for up to six months following administration of the decanoate ester [33–35]. The aforementioned findings suggest that risperidone LAI should be preferred to typical LAIs when the goal is to minimize side effects, whilst combating relapse due to that noncompliance, in psychosis patients with comorbid SUDs. Further research is required to make conclusions about other atypical LAIs for schizophrenia patients with SUDs.

## 6. LAIs for Involuntary Outpatient Commitment

**Figure 1:**
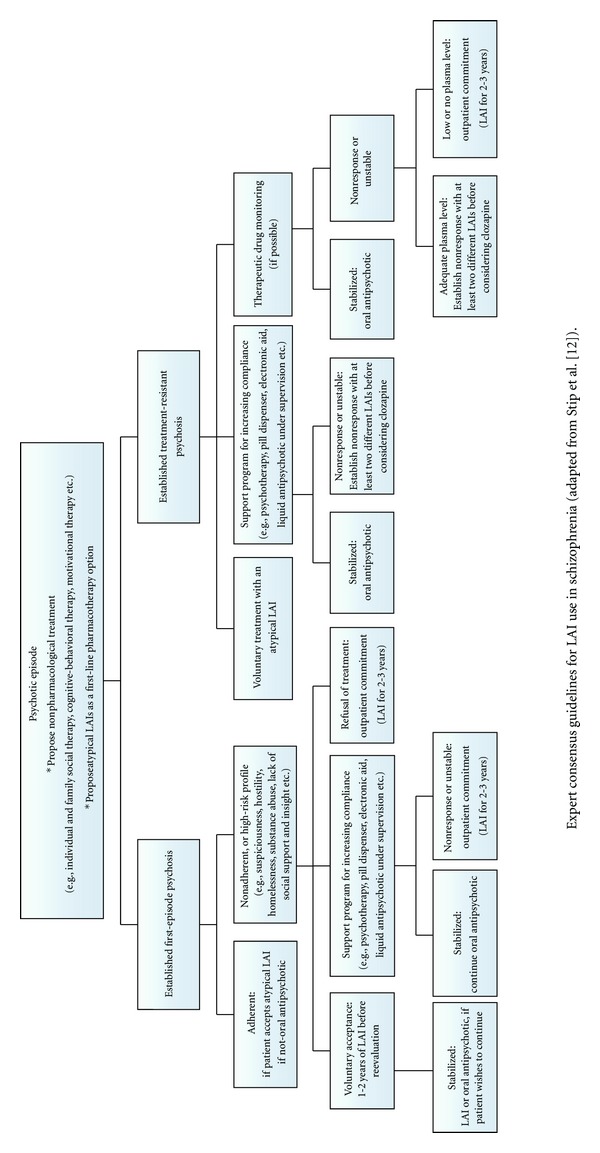


Despite the potential benefits, prescription of LAIs is not a guarantee of compliance. For example, a study by Olfson et al. [86] in California MEDICAID patients prescribed an LAI for poor compliance found that less than 10% of patients continued treatment after the six-month follow-up. This suggests that a legal framing in the form of involuntary outpatient commitment—in conjunction with the help of an interdisciplinary team—can be necessary to ensure the observance of LAIs. In Québec, the order for involuntary outpatient commitment is obtained within via the Supreme Court of Québec for the patients whose capacity to take care of themselves is deteriorated by lack of awareness of illness and difficulty of evaluating the advantages/disadvantages of agreement or refusal of treatment. The purpose of it is to help the patients to control their disease and possibly, to regain control of their life. Even if it appears paradoxical, this coercive step aims at as well as possible guaranteeing the autonomy of the person, especially because lack of insight is an integral symptom of the disease [3]. Moreover, despite obvious concerns that outpatient commitment may negatively affect the therapeutic alliance, our data among 39 schizophrenia patients who explicitly refused treatment revealed that the therapeutic alliance remained unchanged, even after the legal procedure [87]. Recently, an expert consensus panel from the *association des médecins psychiatres du Québec* (AMPQ) has recommended more widespread prescription of LAIs and involuntary outpatient commitment—with the goal of increasing treatment compliance among FEP patients (Figure 1) [12]. In the countries where the regulation of LAIs is more frequent, it is generally less difficult enforce outpatient commitment than in Québec. In Australia, for example, a one-page report of health signed by only one psychiatrist, the “community treatment order”, is enough to obtain authorization to treat a patient without his consent, with the aim of avoiding future deterioration and preventing it when it occurs [88, 89].

In this context, three studies compared oral to depot antipsychotics for schizophrenia patients on involuntary outpatient commitment/community treatment orders [88–90]. One retrospective study found significantly less medication adherence and more rehospitalization among 123 patients on community treatment orders who were receiving oral antipsychotics, compared to those receiving LAIs [88]. Another retrospective study among 94 community treatment orders patients revealed that LAIs were associated with fewer crisis team referrals and other episodes of relapse [89]. In addition, a prospective study found that patients undergoing sustained periods of involuntary outpatient commitment (six months or more) were more likely to remain compliant with medication and other treatment, relative to those who underwent only brief outpatient commitment or none [90]. In that study, administration of depot antipsychotics significantly improved treatment adherence independently of the effect of sustained outpatient commitment. As a whole, these preliminary case-control trials reveal that LAIs may be an important tool to improve outcomes in patients on involuntary outpatient commitment and they highlight the need for better controlled trials to confirm the superior efficacy of LAIs in this population.

## 7. Conclusion

The present systematic review compared LAIs with their oral equivalents in order to avoid bias associated with comparing different molecules in the oral and injectable forms. Randomized studies suggest that not all LAIs are the same; for example, long-acting risperidone may be associated with equal or less side effects than oral risperidone, whereas fluphenazine enanthate and decanoate may be associated with equal or more side effects than oral fluphenazine. These differences may be the result of more predictable drug delivery via the use of microspheres [17, 31]. However, these conclusions are limited because of the difficulty in finding appropriate equivalent doses from oral antipsychotics to LAIs and the wide ranges of equivalent doses noted in different studies [91–93]. In addition, randomized studies suggest that LAIs reduce risk of relapse when combined with additional interventions such as individual and family social therapy and/or a nurse available for home visits. On the other hand, randomized studies may have underestimated the benefits of LAIs because of underrepresentation of the nonadherent patient population. Indeed, large-scale naturalistic studies show major benefits of LAIs versus oral antipsychotics, especially among FEP patients [9, 61]. Among SUD patients, preliminary studies indicate that risperidone LAI may be the preferred compound, possibly due to a lower rate of side effects and interactions with drugs of abuse. Nonetheless, clinicians must be aware of the potential for antipsychotics to interact with psychostimulants, resulting in increased EPS and, in some cases, enhanced drug liking/craving and drug relapse, especially at high doses [75–77]. Preliminary studies also suggest that LAIs may reduce violence in patients with a history of violence and decrease relapse and rehospitalization in schizophrenia patients on involuntary outpatient commitment, relative to oral antipsychotics [56, 88–90].

Despite the potential advantages of LAIs, their use in the United States, Canada, and Germany remains the lowest among developed countries [94]. We feel that the evidence in favor of more widespread use of LAIs in schizophrenia (especially among FEP patients) is mounting, having been boosted by the advent of newer molecules and better methods of delivery. Nevertheless, we acknowledge that the efficacy benefits of LAIs may be constrained by the limitations of antipsychotics themselves. For example, at the end of the Del Giudice et al. [52] study, the chance of experiencing a relapse in patients treated with fluphenazine enanthate returned to the level of those treated with oral fluphenazine. These data indicate that LAIs do not prevent a patient from relapsing, but they may increase the time between relapses by improving adherence. Indeed, it is crucial that the only patients that evidenced sustained improvement on LAIs for over two years was the group who received intensive individual and family social therapy [54]. This finding is a testament to the importance of psychotherapy and quality followup services to maintain the benefits of LAIs [95, 96]. Further research is required to elucidate which special populations may benefit most from LAI therapy and which psychosocial interventions work best when paired with LAIs.

## Figures and Tables

**Table 1:** Characteristics of some older and newer LAIs [12, 17, 19].

	Vehicle	Preparation	Dosing interval	Injection site pain/reactions	Comments
Flupenthixol	Ester (decanoate)	Viscoleo	2–4 w	^+ + +^[20, 21]	—
Fluphenazine	Ester (decanoate)	Sesame seed oil	2–5 w	^+ + +^[20, 21]	—
Haloperidol	Ester (decanoate)	Sesame seed oil	4 w	^+ + +^[20–22]	—
Zuclopenthixol	Ester (decanoate)	Viscoleo	2–4 w	^+ + + +^[20, 21]	—
Paliperidone	Ester (palmitate)	Water (nanosuspension)	4 w	^+ +^[23]	Metabolite of risperidone
Olanzapine	Salt (pamoate)	Water (microcrystalline suspension)	2–4 w	^+ +^[24, 25]	Monitoring required for at least three hours due to small risk of IAIV
Risperidone	Microspheres	Water	2 w	^+^[23, 26, 27]	Refrigeration required

IAIV: inadvertent intravascular injection; ^+^: minimal; ^+ +^: low; ^+ + +^: moderate; ^+ + + +^: high.

**Table 2:** Randomized and naturalistic studies comparing LAIs with their oral equivalents.

Study	*N*	Duration	In/out, patient	Design	Antipsychotic dose	Dropouts *Relapse *	Dropouts *Side effects *	Comments
Zuardi et al. [47]	22	16 weeks	In	RDM DB	HAL DEC 20 mg IM/4 w : 1 mg/d PO*	ND	ND	LAI = marginally better efficacy and more EPS
Kinross-Wright and Charalampous [48]	40	6 weeks	In	RDM DB	FLZ ETH^#^ 25 mg IM/2 w versus 2.5–7.5 mg PO	ND	ND	—
Ravaris et al. [49]	39	24 weeks	In	RDM DB	FLZ ETH 12–25 mg IM/2 w versus 2.5–10 mg PO	5% (LAI) 0% (PO)	5% (LAI) 6% (PO)	—
Haider [50]	43	6 weeks	In	RDM DB	FLZ ETH 25 mg IM/2-3 w versus 2.5–10 mg/d PO	ND	ND	LAI = more EPS
van Praag et al. [51]	50	4 weeks	In	RDM DB	FLZ ETH^#^ 25 mg IM/3 w versus 7 mg/d PO	ND	ND	LAI = more EPS
Del Giudice et al. [52]	88	15 months	Out	RDM SB^P^	FLZ ETH^#^ 25 mg IM/2 w versus 22 mg/d PO	ND	ND	LAI = longer time to relapse and more EPS
Rifkin et al. [53]	73	12 months	Out	RDM DB	FLZ DEC 12.5 mg IM/2 w: 5 mg/d PO^+^	4% (LAI) 7% (PO)	22% (LAI) 4% (PO)	LAI = more EPS
Hogarty et al. [54]	105	24 months	Out	RDM DB	FLZ DEC 34–43 mg IM/2 w versus 10–12 mg/d PO	23% (LAI^+ST^) 50% (LAI) 55% (PO) 66% (PO^+ST^)	9% (LAI) 0% (PO)	LAI = more anxiety/depression, but less positive symptoms
Schooler et al. [55]	214	12 months	Out	RDM DB	FLZ DEC^#^ 34 mg IM/3 w versus 25 mg/d PO	24% (LAI) 33% (PO)	5% (LAI) 4% (PO)	—
Arango et al. [56]	46	12 months	Out	RDM OL	ZUC DEC^#^ 233 mg IM/2 w versus 35 mg/d PO	4% (LAI) 5% (PO)	ND	LAI = less violence
Chue et al. [26]	541	3 months	Both	RDM DB	RIS MIC 25–75 mg IM/2 w versus 2–6 mg/d PO	4% (LAI) 3% (PO)	6% (LAI) 5% (PO)	LAI = less prolactin elevation
Bai et al. [57]	50	12 months	In	RDM SB^I^	RIS MIC 25–50 mg IM/2 w versus 4–6 mg/d PO	8% (LAI) 0% (PO)	4% (LAI) 0% (PO)	LAI = lower UKU score, lower EPS and prolactin levels
Eli Lily [58]	524	24 months	Out	RDM OL	OLZ PAM 150–405 mg IM/4 w versus 5–20 mg/d PO	16% (LAI) 10% (PO)	10% (LAI) 10% (PO)	LAI = less rehospitalisations
Kane et al. [25]	1065	6 months	Out	RDM DB	OLZ PAM 45 mg IM/4 w versus 150 mg IM/2 w versus 405 mg IM/4 w versus 300 mg IM/2 w versus 10, 15, 20 mg/d PO	6% (LAI^HI^) 13% (LAI^MD^) 19% (LAI^LO^) 8% (PO)	3% (LAI^HI^) 3% (LAI^MD^) 5% (LAI^LO^) 3% (PO)	—
Kim et al. [60]	50	24 months	Out	NAT (FEP)	RIS MIC^#^ 29 mg/2 w versus 3 mg/d PO	23% (LAI) 75% (PO)	ND	LAI = lower relapse rate
Zhu et al. [59]	299	12 months	Out	NAT	HAL DEC^#^ 100 mg/4 w versus 11 mg/d PO FLZ DEC^#^ 25 mg/2 w versus 12 mg/d PO	ND	ND	LAI = longer time to discontinuation
Tiihonen et al. [61]	2230	3.6 years^#^	Out	NAT (FEP)	PER DEC versus oral equivalent	ND	ND	LAI = lower risk of rehospitalization
Tiihonen et al. [9]	2588	24 months	Out	NAT (FEP)	RIS MIC, HAL DEC, PER DEC, ZUC DEC versus oral equivalent	ND	ND	LAI = lower risk of rehospitalization

In = inpatients; Out = outpatients; # = mean; * = ratio; +ST = fluphenazine decanoate + social therapy;  : = ratio; FEP = first-episode psychosis; UKU = Udvalg for Kliniske Undersøgelser Side Effect Rating Scale; LAI = long-acting injectable antipsychotic; PO = oral equivalent; NAT = naturalistic design; RDM = randomized; DB = double blind; SB^I^ = investigator blind; SB^P^= patient blind; OL = open label; ND = no usable data; FLZ DEC = fluphenazine decanoate; FLZ ETH = fluphenazine enanthate; ZUC DEC = zuclopenthixol decanoate; HAL DEC = haloperidol decanoate; PER DEC = perphenazine decanoate; RIS MIC = risperidone microspheres; OLZ PAM = olanzapine pamoate; HI = high dose; MD = medium dose; LO = low dose; + = the typical patient was treated with 10–20 mg oral fluphenazine.
